# Supplementary material for: Redox and Nucleophilic Reactions of Naphthoquinones with Small Thiols and Their Effects on Oxidization of H2S to Inorganic and Organic Hydropolysulfides and Thiosulfate
Source: Int J Mol Sci. 2023 Apr 19;24(8):7516. doi: 10.3390/ijms24087516 (PMC10138938; doi:10.3390/ijms24087516)
Supplement: Supplementary file 1 [file ijms-24-07516-s001.zip › Olson et al supplemental info.pdf]

Supplemental Information

Redox and Nucleophilic Reactions of Naphthoquinones with Small Thiols and Their Effects on Oxidization of H_2S to Inorganic and Organic Hydropolysulfides and Thiosulfate

Kenneth R. Olson[1,2,*] Kasey Clear[3], Yan Gao[1], Zhilin Ma[1,2], Nathaniel M. Cieplik[1,2], Alyssa R. Fiume[1,2], Dominic J. Gaziano[1,2], Stephen M. Kasko[1,2], Jennifer Luu[1,2], Ella Pfaff[1,2], Anthony Travos[1,2], Cecilia Velander[1,2], Katherine J. Wilson[1,2], Elizabeth D. Edwards[3], Karl D. Straub[4,5], Gang Wu[6]

1. Indiana University School of Medicine - South Bend Center, South Bend, Indiana 46617 USA
2. Department of Biological Sciences, University of Notre Dame, Notre Dame, IN 46556, USA
3. Department of Chemistry and Biochemistry, Indiana University South Bend, South Bend, Indiana 46615 USA
4. Central Arkansas Veteran's Healthcare System, Little Rock, AR 72205, USA
5. Departments of Medicine and Biochemistry, University of Arkansas for Medical Sciences, Little Rock, AR 72202, USA
6. Department of Internal Medicine, the University of Texas – McGovern Medical School at Houston, Houston, TX 77030, USA.

***Address correspondence to:**

Kenneth R. Olson, Ph.D.
Indiana University School of Medicine -South Bend
Raclin Carmichael Hall
1234 Notre Dame Avenue
South Bend, IN 46617
Phone: (574) 631-7560
Fax: (574) 631-7821
e-mail: olson.1@nd.edu

Supplemental Figures

Supplemental Figure S1. Effects of 0.1 µM superoxide dismutase (SOD) or 1 µM catalase (Cat) on inorganic and organic sulfur compounds produced by incubation of 10 µM 1,4-NQ and 300 µM H_2S for 10 min. Both enzymes decreased hydropersulfide and hydropolysulfide production but did not affect the monothiols.

Supplemental Figure S2. Effects of GSH, Cys and propylamine (PA) on SSP4 fluorescence. (**A**) Fluorescence produced by addition of 10 µM of the mixed H_2S/polysulfide, K_2S_n (n=1-5) to 10 µM of SSP4 is unaffected when 1 mM GSH is added 2 hours after SSP4 and K_2S_n, but completely inhibited when SSP4, K_2S_n and GSH are added simultaneously. Both GSH (**B**) and Cys (**C**) concentration-dependently decrease SSP4 fluorescence when added simultaneously to SSP4 and K_2S_n either without (0 µM 1,4-NQ) or with 1,4-NQ (10 µM 1,4-NQ). (**D**) 1,4-NQ (10 µM) does not metabolize polysulfides (K2S_n, 20 µM). K_2S_n was bubbled with air for 20 min to remove H_2S and SSP4 (5 µM) added at 0, 10, 30 and 60 min to wells containing either K_2S_n alone (white bars) or K_2S_2 plus 1,4-NQ (black bars). Mean +SEM, n=4 wells per treatment; **, $p<0.01$; ***, $p<0.001$ vs control. (**E**) 1 mM PA nearly halves fluorescence from SSP4 and 10 µM K_2S_n when added after or 10 min before K_2S_n but only by 20% when added 2 h after K_2S_n. GSH (1 mM) completely inhibits SSP4 and 10 µM K_2S_n unless it is added 2 h after PA and K_2S_n. Mean +SEM, n=4 wells per treatment; **, $p<0.01$; ***, $p<0.001$ vs K_2S_n.

Supplemental Figure S3. GSH and Cys directly interfere with thiosulfate detection by Ag-nanoparticles (AgNPs). (**A**) Effects of 0.1 µM superoxide dismutase (SOD) and 1 µM catalase (Cat), alone or in combination, on thiosulfate production from 300 µM H_2S in the absence (H_2S

only) or presence of 10 μM NQ, 1 mM glutathione (+GSH) or 1 mM cysteine (+Cys). With H_2S alone, TS production was decreased by Cat and slightly increased by SOD+Cat. With H_2S plus NQ, TS was greatly increased by SOD, decreased by Cat but unaffected by SOD+Cat. There was essentially no TS production in the presence of GSH without or with SOD or Cat, and only minimal production in the presence of Cys. Inset shows a typical thiosulfate standard curve. (**B-D**) Effects of GSH and Cys on thiosulfate detection by AgNPs. (**B**) Increasing concentrations of thiosulfate (TS) were added to AgNPs followed by addition of 1 mM GSH or 1 mM Cys. Detection of TS was almost completely inhibited by GSH, whereas more TS was measured than expected in the presence of all but 300 μM Cys. (**C**) Little TS was detected when TS was added to GSH or Cys prior to addition of AgNPs. (**D**) Effects of variable GSH concentrations on AgNP measurement of 100 μM TS when GSH was added simultaneously to TS-AgNP (t=0) or 10 min later (t=10). Delaying GSH addition increased thiosulfate detection at higher GSH concentrations. (**E**) Effects of 1 mM GSH and 1 mM Cys on thiosulfate production from 10 μM 1,4-NQ and 300 μM H_2S measured by the HPLC-MBB method. **A-D**; mean +SEM, n=8 wells per treatment; *, p <0.05; ***, p <0.001 compared to H_2S from same treatment. a, reported previously in [1]; **E**; mean, n=2.

Supplemental Figure S4. EPR spectra of various combinations of GSH and Cys with 1,4-NQ. (**A**) The EPR spectrum was silent after addition of 4 mM 1,4-NQ to 4 mM GSH (1:1 GSH:1,4-NQ) ***(a)***, but appeared when solution was diluted with 4 mM 1,4-NQ to a 0.25:1 GSH:1n4-NQ ratio ***(b)***. (**B**) There was no obvious spectrum with 4 mM 1,4-NQ and 4 mM Cys (1:1 Cys:1,4-NQ ***(a)***). Addition of 2 vol. of 4 mM 1,4-NQ to 1 vol. of ***a*** (0.3:1 Cys:1,4-NQ) produced the characteristic 1,4-NQ semiquinone (***(b)***). Only a faint semiquinone spectrum was produced

when 0.7 vol. of 4 mM 1,4-NQ was added to volume of ***a***, (0.6:1 Cys:1,4-NQ, ***(c)***), whereas the spectrum was restored when the Cys was further diluted with 1,4-NQ (0.3:1 Cys:1,4-NQ, ***(d)***).

Supplemental Figure S5. EPR spectra of GSH, Cys and H_2S reactions with 4 mM juglone.

Supplemental Figure S6. EPR spectra of GSH, Cys and H_2S reactions with 4 mM plumbagin.

Supplemental Figure S7. EPR spectra of GSH, Cys and H_2S reactions with 4 mM 2-methoxy-1,4 NQ (2-MNQ).

Supplemental Figure S8. Typical traces of oxygen consumption during formation of 300 μM GSH or Cys adducts with 30 μM NQs and the effects of 0.1 μM SOD.

Supplemental Figure S9. Effects of propylamine (PA) reactions with 1,4-NQ, H_2S and GSH on oxygen consumption. PA (**A**) and 1,4-NQ (**B**) concentration-dependently increased oxygen consumption when added to the other. (**C**) Short-term (15 min) incubation of 100 μM 1,4-NQ with 100 μM or 500 μM PA did not affect oxygen consumption when 100 μM of either GSH or Cys are added. (**D**) Incubation of 5 mM 1,4-NQ with 5 mM PA for 3 h then dilution to 100 μM, 500 μM and 1.38 mM nearly completely prevented oxygen consumption when equimolar (100 μM, 500 μM and 1.38 mM) GSH were added. (**E**) PA (30 μM) did not affect oxygen consumption when added to 300 μM H_2S, 30 μM 1,4-NQ plus H_2S or 1,4-NQ plus H_2S plus 1 mM GSH. (**F**) Incubation of 30 μM 1,4-NQ with 1 mM PA (+PA) or without PA (-PA) did not affect oxygen consumption by 1,4-NQ and 300 μM H_2S with 1 mM GSH.

Supplemental Figures S10-S13. Polysulfide production (SSP4 fluorescence) from H_2S catalyzed by NQ-GSH and NQ-Cys adducts. Adducts were prepared by incubating 1 mm juglone (**S9**), plumbagin (**S10**), menadione (**S11**) or 2-MNQ (**S12**) with 1 mM GSH (**A,E**), 1 mM Cys (**B,F**), 1 mM PA (**C,G**) or 1 mM H_2S (**D,H**) for 1 h then diluting the adduct to 10 µM or 30 µM and reacting it with either 100 µM or 300 µM H_2S. Bar graphs summarize effects at 90 min, mean +SE, n=4 wells; *, *p<0.5*; **, *p<0.01*; ***, *p<0.001* vs NQ without adduct.

Supplemental Figure S14. Polysulfide production (SSP4 fluorescence) from 10 µM NQs, and 300 µM H_2S after addition of 10 µM GSH (**A**) or 10 µM Cys (**B**). Bar graphs summarize effects at 100 min, mean +SE, n=4 wells; *, *p<0.5*; **, *p<0.01*; ***, *p<0.001* vs NQ without GSH or Cys. (**C**, **D**) Extended time course of polysulfide production (SSP4 fluorescence) by incubation of various concentrations of H_2S with 10 µM menadione (**C**) or 2-MNQ (**D**), mean +SE, n=4 wells per treatment.

Supplemental Figure S15. Characterization of bovine serum albumin (BSA) and 1,4-NQ adducts and their effects on H_2S metabolism. (**A-C**) Full- and expanded-scale absorbance spectra of 100 µM 1,4-NQ and 100 µM BSA. Unmixed (black line) shows individual spectra in parallel cuvettes, mixed (red line) spectra after mixing BSA and 1,4-NQ. Numbered arrows indicate peaks. (**D**) EPR spectra of 4 mM 1,4-NQ added to 0.6 mM BSA or 2.9 mM hemoglobin (Hb). Both spectra are characteristic of the 1,4-NQ semiquinone radical. (**E**) BSA concentration-dependently inhibited fluorescence when added 2 h after mixing 5 µM SSP4 with 10 µM polysulfide (K_2S_n). Pink bar shows typical BSA concentrations in plasma. (**F**) BSA had only minor effects on fluorescence when added 2 h after mixing 25 µM AzMC with 300 µM

H_2S. (**G**) Effects of BSA on 1,4-NQ metabolism. BSA (100 µM) and 1,4-NQ (100 µM) were incubated for 30 min then diluted to 10 µM BSA/1,4-NQ. H_2S (300 µM) was added to the BSA/1,4-NQ mixture and to 10 µM BSA, 10 µM 1,4-NQ, or PBS buffer and incubated another 30 before addition of AzMC, fluorescence was measured 10 min later. A decrease in H_2S/AzMC fluorescence compared to H_2S in buffer indicates more H_2S was consumed. (**E**, **F**) Mean +SE, n=4 wells per treatment; *, $p<0.5$; ***, $p<0.001$ vs 0 µM BSA. (**G**) Mean +SE, n=8 wells per treatment; ***, $p<0.001$ vs +H_2S. (**H**) Three overlapping traces showing that oxygen consumption is not affected by BSA (100 µM), 1,4-NQ (100 µM) or when 100 µM BSA is added to 100 µM 1,4-NQ.

1. Olson, K. R.; Clear, K. J.; Derry, P. J.; Gao, Y.; Ma, Z.; Cieplik, N. M.; Fiume, A.; Gaziano, D. J.; Kasko, S. M.; Narloch, K.; Velander, C. L.; Nwebube, I.; Pallissery, C. J.; Pfaff, E.; Villa, B. P.; Kent, T. A.; Wu, G.; Straub, K. D., Naphthoquinones Oxidize H(2)S to Polysulfides and Thiosulfate, Implications for Therapeutic Applications. *Int J Mol Sci* **2022,** 23, (21).
