# Supplementary material for: Redox and Nucleophilic Reactions of Naphthoquinones with Small Thiols and Their Effects on Oxidization of H2S to Inorganic and Organic Hydropolysulfides and Thiosulfate
Source: Int J Mol Sci. 2023 Apr 19;24(8):7516. doi: 10.3390/ijms24087516 (PMC10138938; doi:10.3390/ijms24087516)
Supplement: Supplementary file 1 [file ijms-24-07516-s001.zip › Supplemental Figs.pptx]

## Slide 1
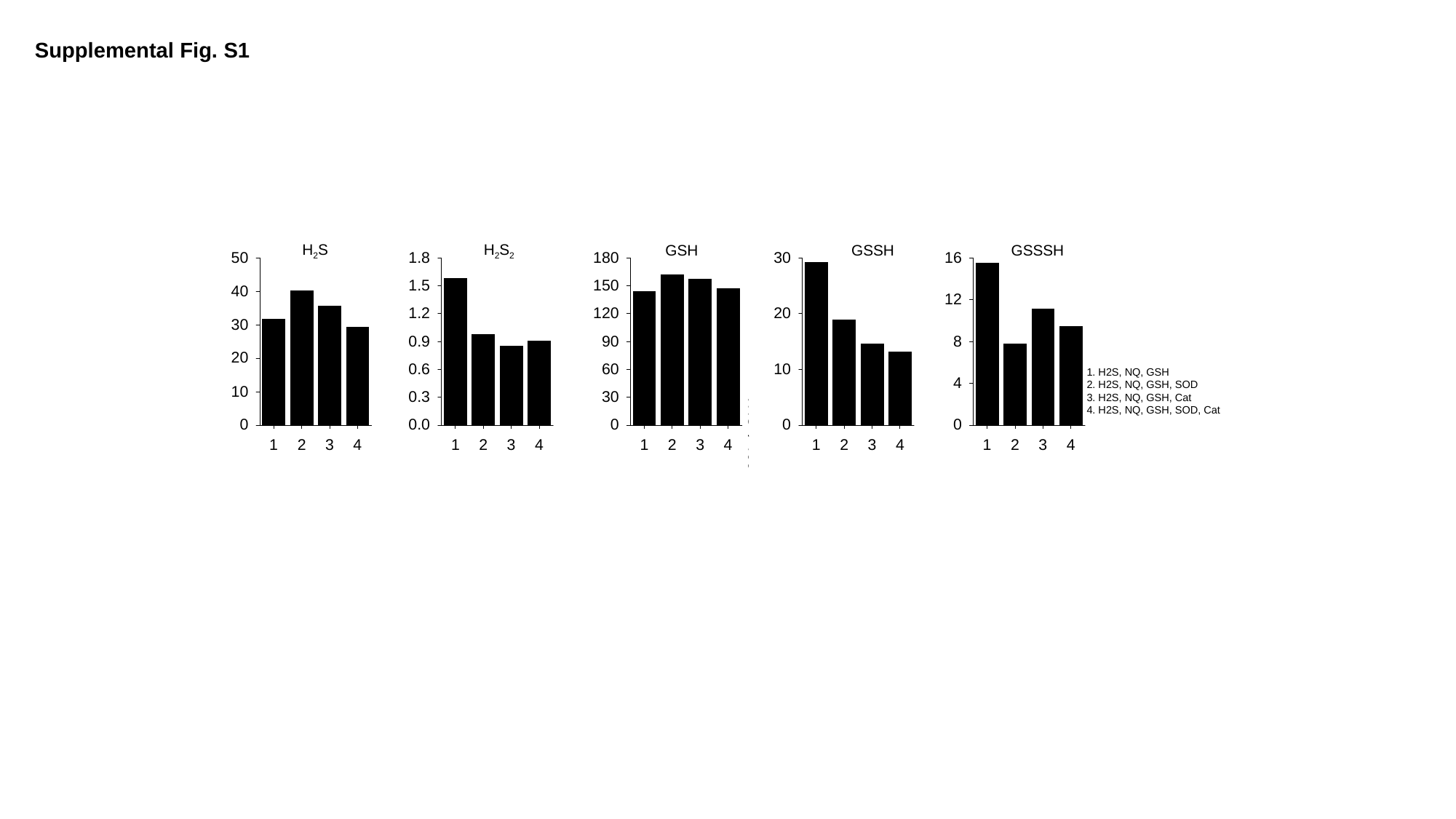

Supplemental Fig. S1
H2S
H2S2
GSH
GSSH
GSSSH
1. H2S, NQ, GSH
2. H2S, NQ, GSH, SOD
3. H2S, NQ, GSH, Cat
4. H2S, NQ, GSH, SOD, Cat

## Slide 2
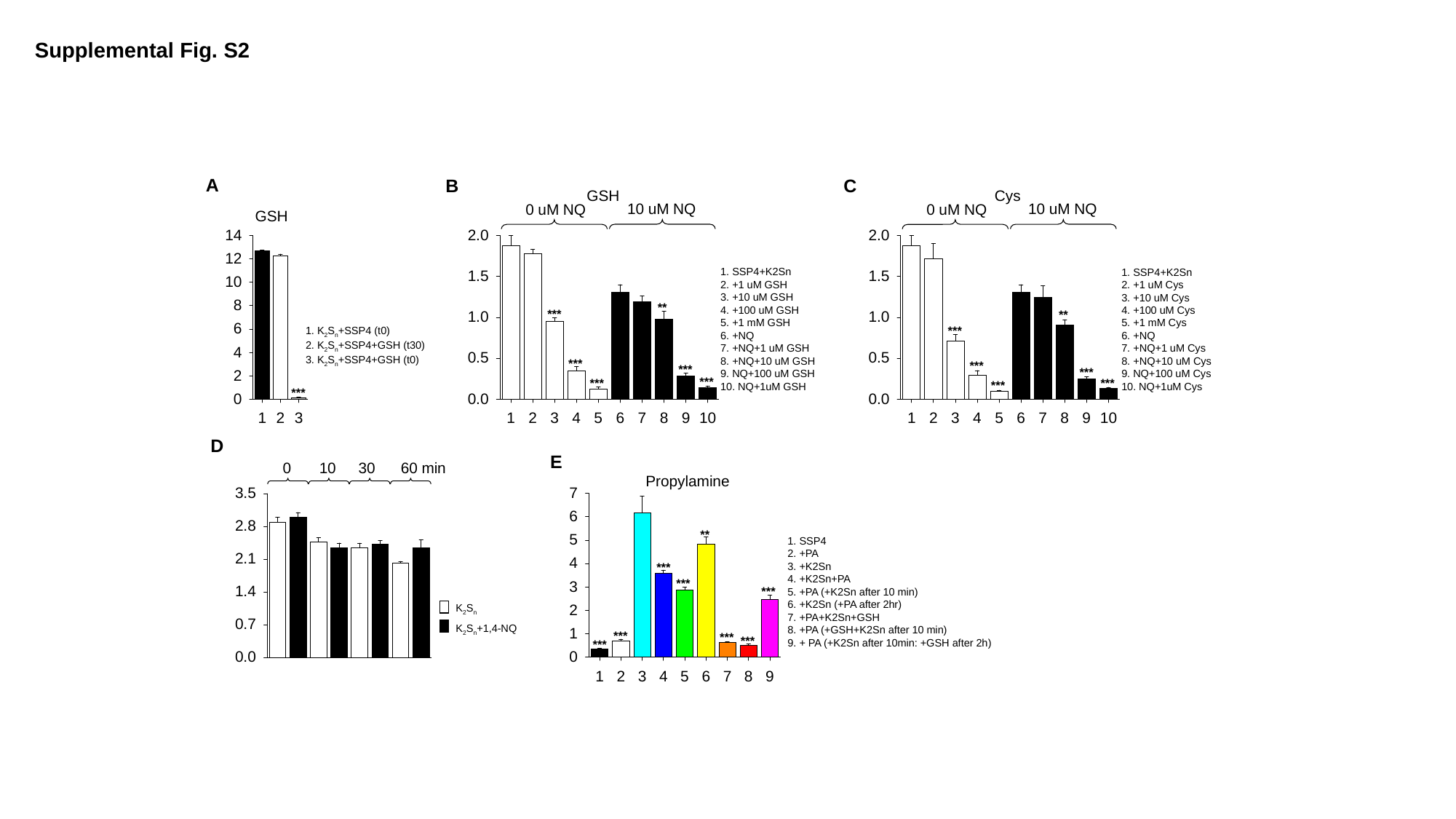

Supplemental Fig. S2
A
C
B
GSH
Cys
10 uM NQ
10 uM NQ
0 uM NQ
1. SSP4+K2Sn
2. +1 uM Cys
3. +10 uM Cys
4. +100 uM Cys
5. +1 mM Cys
6. +NQ
7. +NQ+1 uM Cys
8. +NQ+10 uM Cys
9. NQ+100 uM Cys
10. NQ+1uM Cys
0 uM NQ
GSH
1. SSP4+K2Sn
2. +1 uM GSH
3. +10 uM GSH
4. +100 uM GSH
5. +1 mM GSH
6. +NQ
7. +NQ+1 uM GSH
8. +NQ+10 uM GSH
9. NQ+100 uM GSH
10. NQ+1uM GSH
1. K2Sn+SSP4 (t0)
2. K2Sn+SSP4+GSH (t30)
3. K2Sn+SSP4+GSH (t0)
***
**
***
**
***
***
***
***
***
***
***
***
***
D
0
10
30
60 min
K2Sn
K2Sn+1,4-NQ
**
1. SSP4
2. +PA
3. +K2Sn
4. +K2Sn+PA
5. +PA (+K2Sn after 10 min)
6. +K2Sn (+PA after 2hr)
7. +PA+K2Sn+GSH
8. +PA (+GSH+K2Sn after 10 min)
9. + PA (+K2Sn after 10min: +GSH after 2h)
***
***
***
***
***
***
***
E
Propylamine

## Slide 3
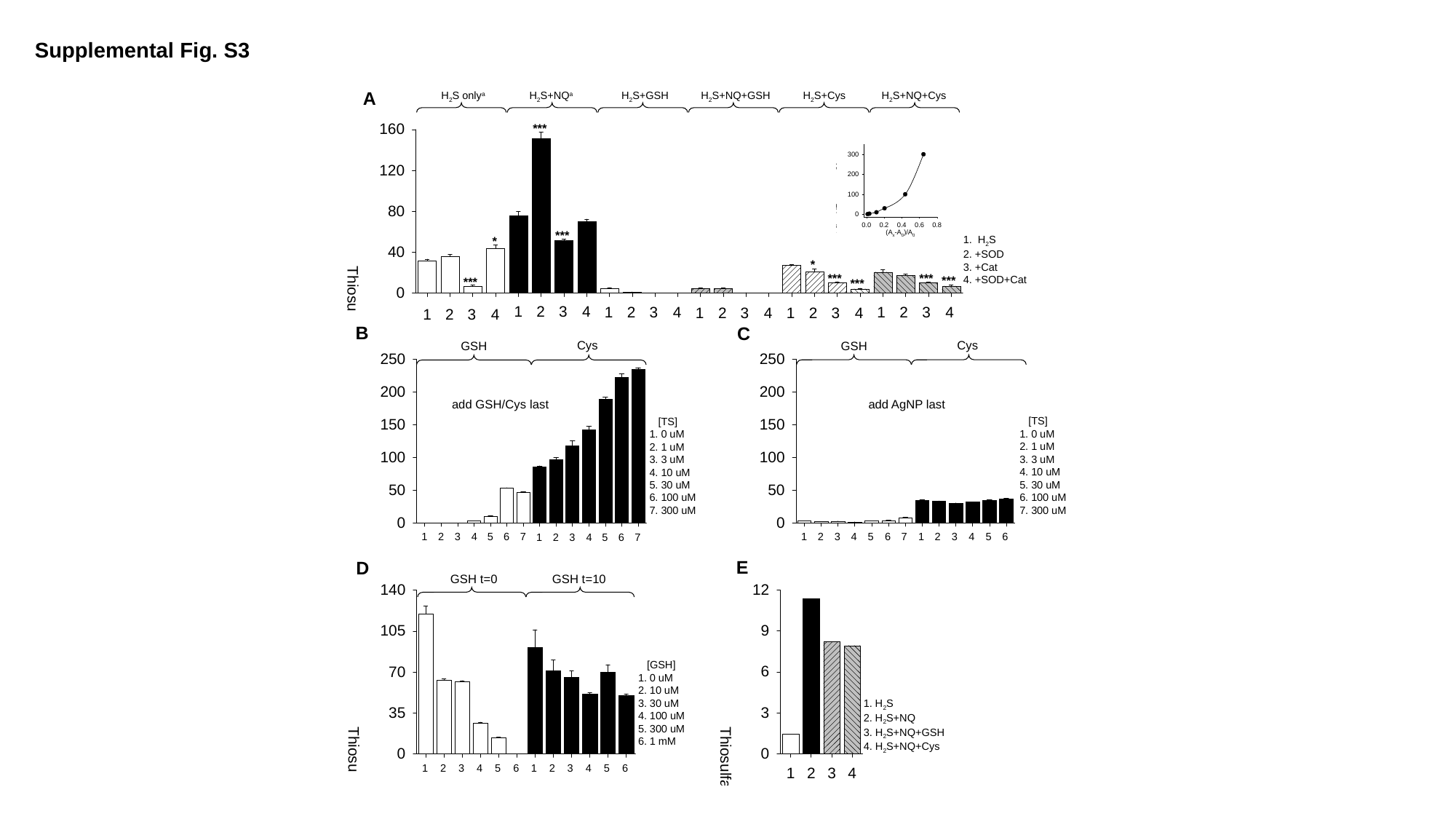

Supplemental Fig. S3
H2S onlya
H2S+NQa
H2S+GSH
H2S+NQ+GSH
H2S+Cys
H2S+NQ+Cys
1. H2S
2. +SOD
3. +Cat
4. +SOD+Cat
1
2
3
4
1
2
3
4
1
2
3
4
1
2
3
4
1
2
3
4
1
2
3
4
A
***
***
*
*
***
***
***
***
***
B
C
Cys
GSH
Cys
GSH
add GSH/Cys last
add AgNP last
 [TS]
1. 0 uM
2. 1 uM
3. 3 uM
4. 10 uM
5. 30 uM
6. 100 uM
7. 300 uM
 [TS]
1. 0 uM
2. 1 uM
3. 3 uM
4. 10 uM
5. 30 uM
6. 100 uM
7. 300 uM
1
2
3
4
5
6
7
1
2
3
4
5
6
1
2
3
4
5
6
7
1
2
3
4
5
6
7
GSH t=10
GSH t=0
 [GSH]
1. 0 uM
2. 10 uM
3. 30 uM
4. 100 uM
5. 300 uM
6. 1 mM
1
2
3
4
5
6
1
2
3
4
5
6
E
D
1. H2S
2. H2S+NQ
3. H2S+NQ+GSH
4. H2S+NQ+Cys

## Slide 4
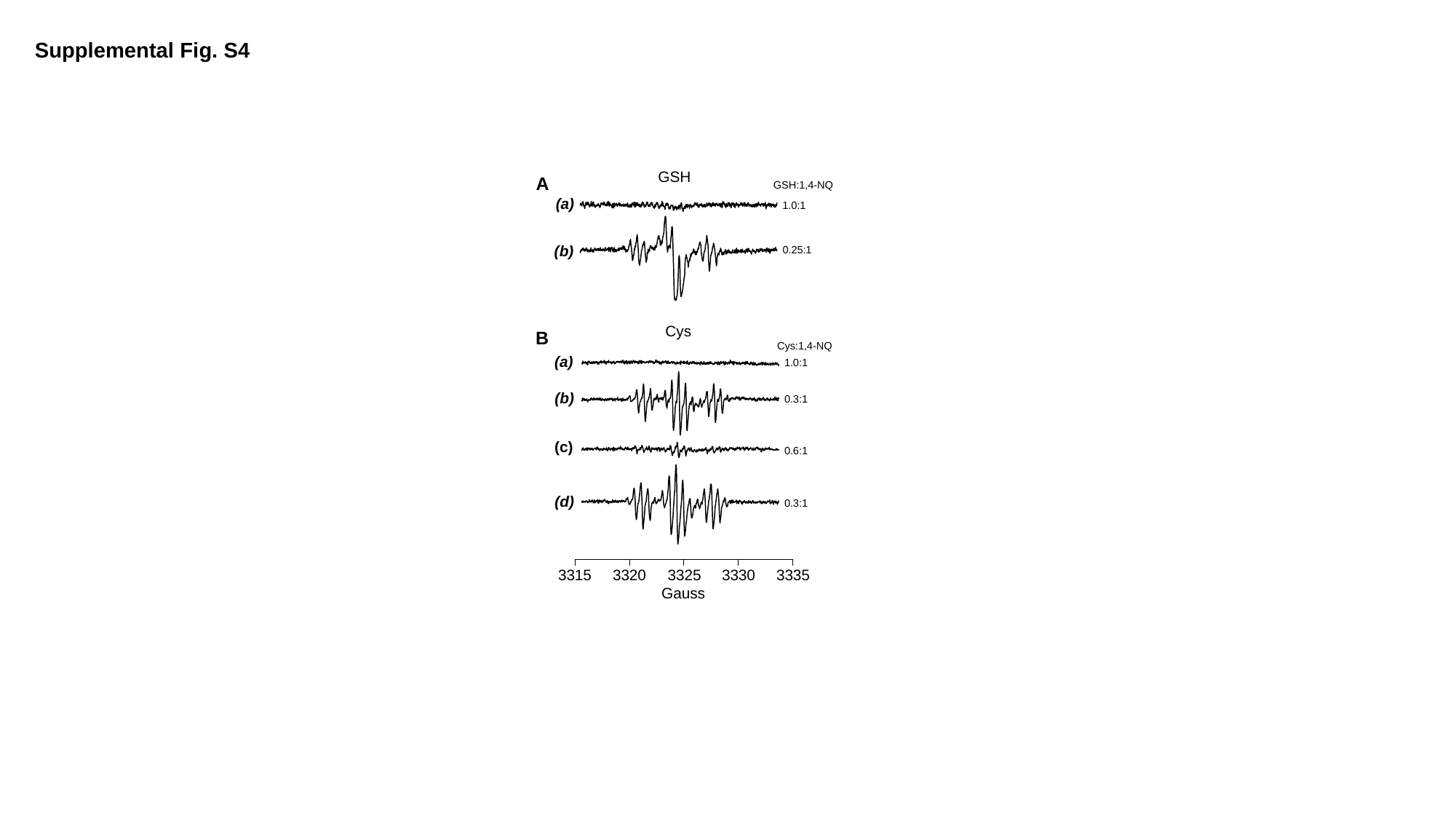

Supplemental Fig. S4
GSH
A
(a)
1.0:1
(b)
0.25:1
1.0:1
0.3:1
0.6:1
0.3:1
(a)
(b)
(c)
(d)
Cys
B
GSH:1,4-NQ
Cys:1,4-NQ
3315
3320
3325
3330
3335
Gauss

## Slide 5
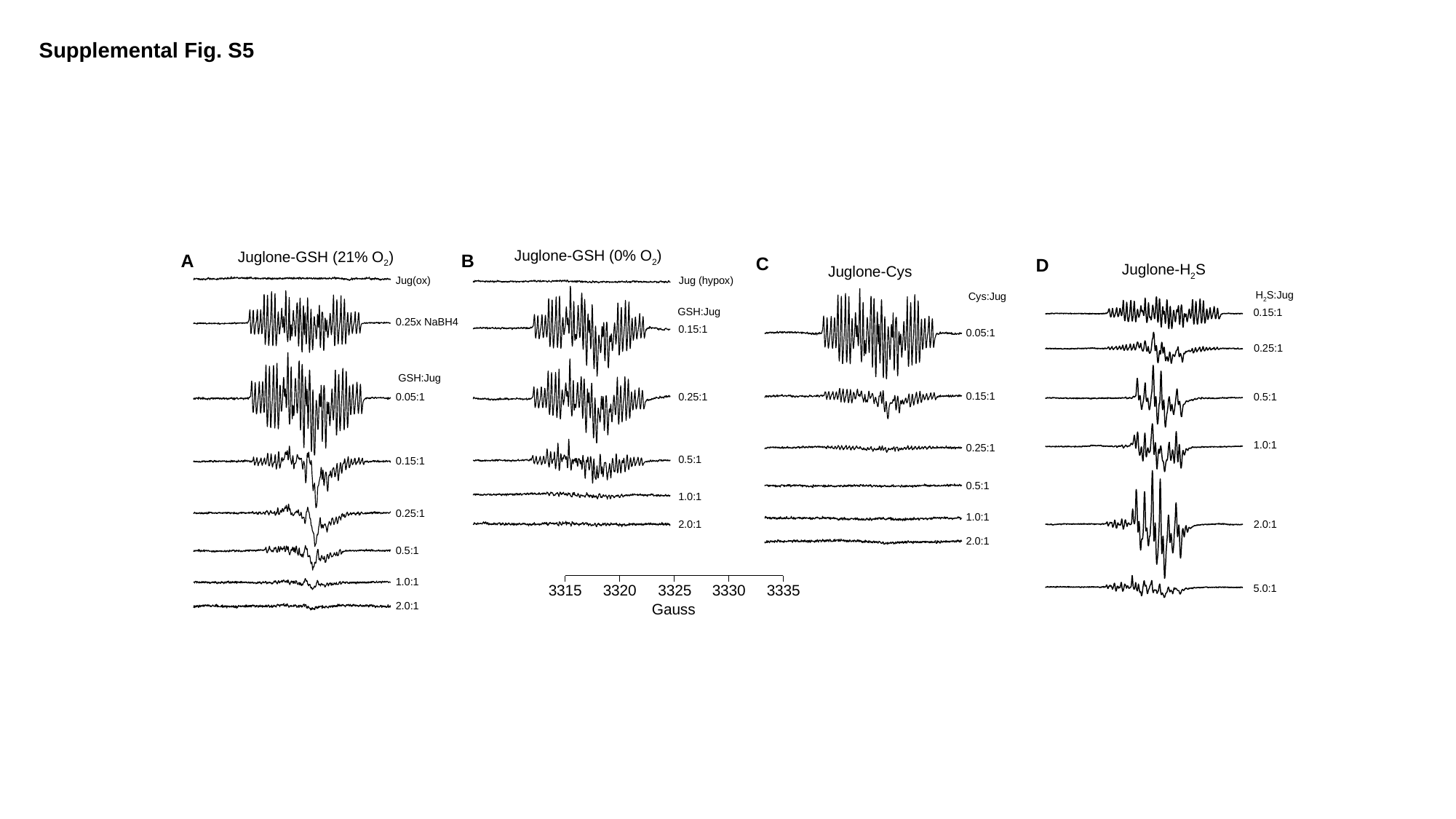

Supplemental Fig. S5
Juglone-GSH (21% O2)
Jug(ox)
0.25x NaBH4
0.05:1
0.15:1
0.25:1
0.5:1
1.0:1
2.0:1
Jug (hypox)
0.15:1
0.25:1
0.5:1
1.0:1
2.0:1
D
Juglone-H2S
0.15:1
0.25:1
0.5:1
1.0:1
2.0:1
5.0:1
Juglone-Cys
0.05:1
0.15:1
0.25:1
0.5:1
1.0:1
2.0:1
Juglone-GSH (0% O2)
B
A
C
H2S:Jug
Cys:Jug
GSH:Jug
GSH:Jug
3315
3320
3325
3330
3335
Gauss

## Slide 6
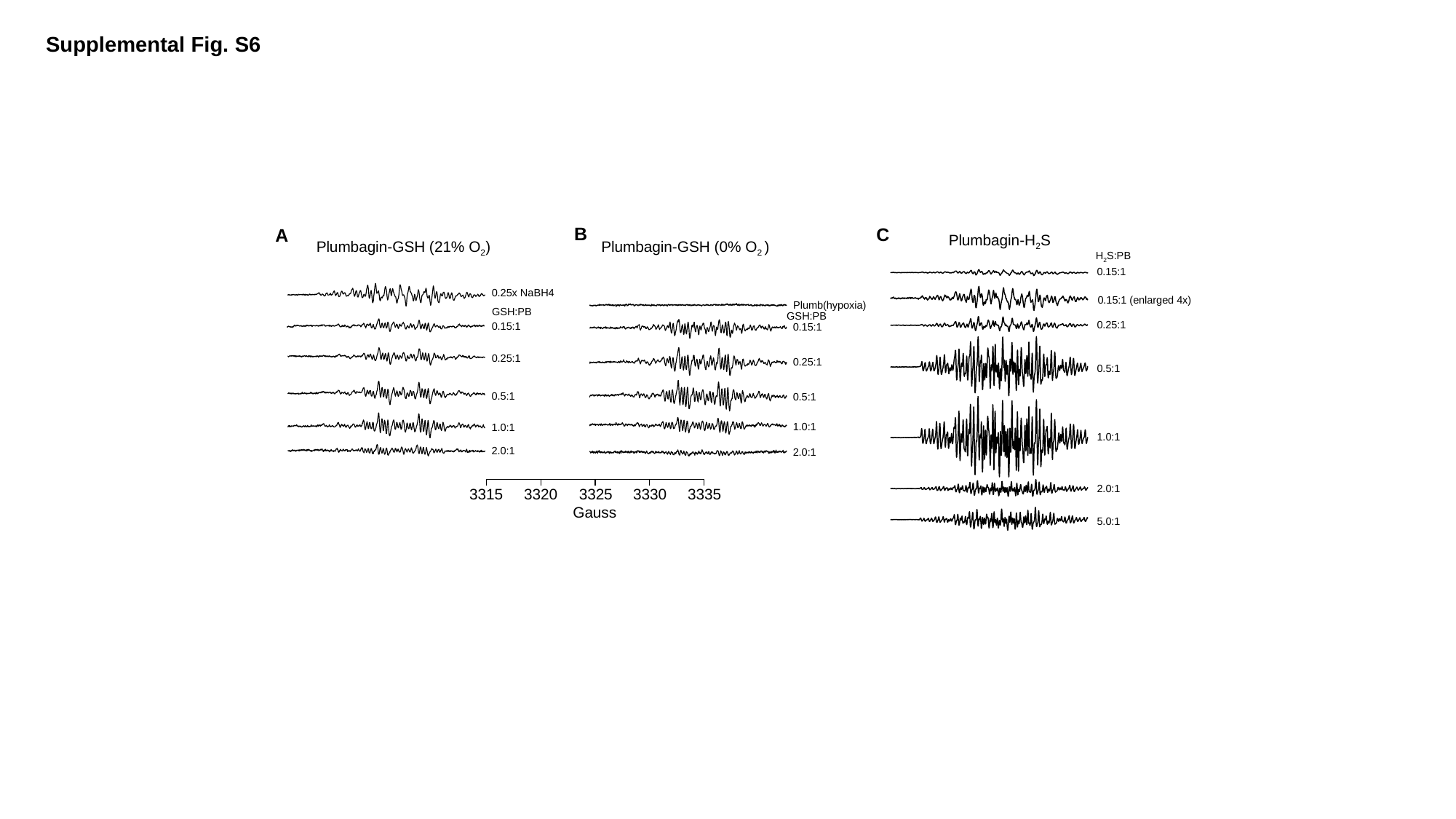

Supplemental Fig. S6
0.15:1
0.15:1 (enlarged 4x)
0.25:1
0.5:1
1.0:1
2.0:1
5.0:1
Plumbagin-GSH (21% O2)
0.25x NaBH4
0.15:1
0.25:1
0.5:1
1.0:1
2.0:1
Plumb(hypoxia)
0.15:1
0.25:1
0.5:1
1.0:1
2.0:1
B
C
A
Plumbagin-H2S
Plumbagin-GSH (0% O2 )
H2S:PB
GSH:PB
GSH:PB
3315
3320
3325
3330
3335
Gauss

## Slide 7
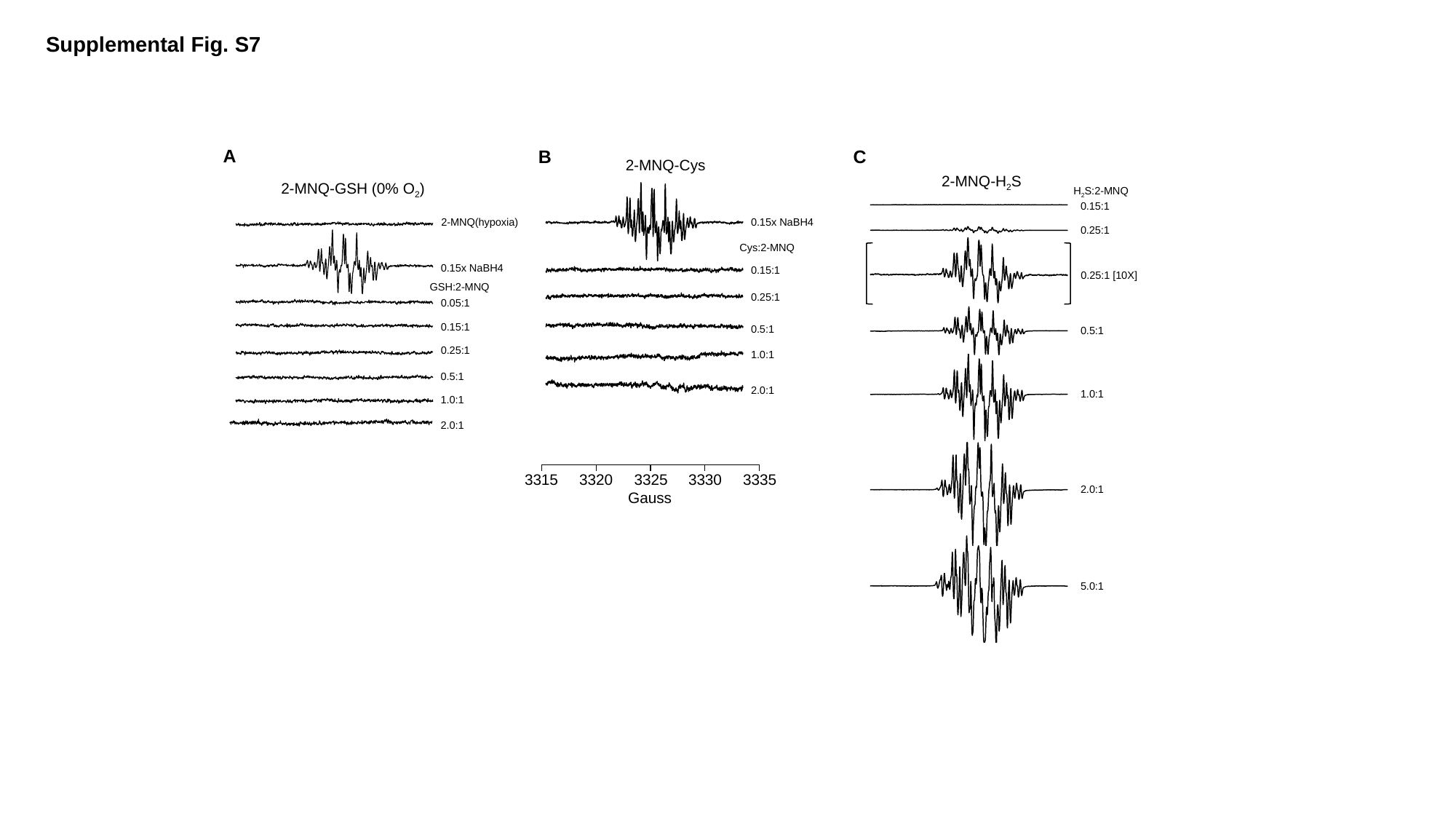

Supplemental Fig. S7
0.15:1
0.25:1
0.25:1 [10X]
0.5:1
1.0:1
2.0:1
5.0:1
2-MNQ-H2S
0.15x NaBH4
0.15:1
0.25:1
0.5:1
1.0:1
2.0:1
2-MNQ-Cys
2-MNQ(hypoxia)
0.15x NaBH4
0.05:1
0.15:1
0.25:1
0.5:1
1.0:1
2.0:1
2-MNQ-GSH (0% O2)
A
C
B
H2S:2-MNQ
Cys:2-MNQ
GSH:2-MNQ
3315
3320
3325
3330
3335
Gauss

## Slide 8
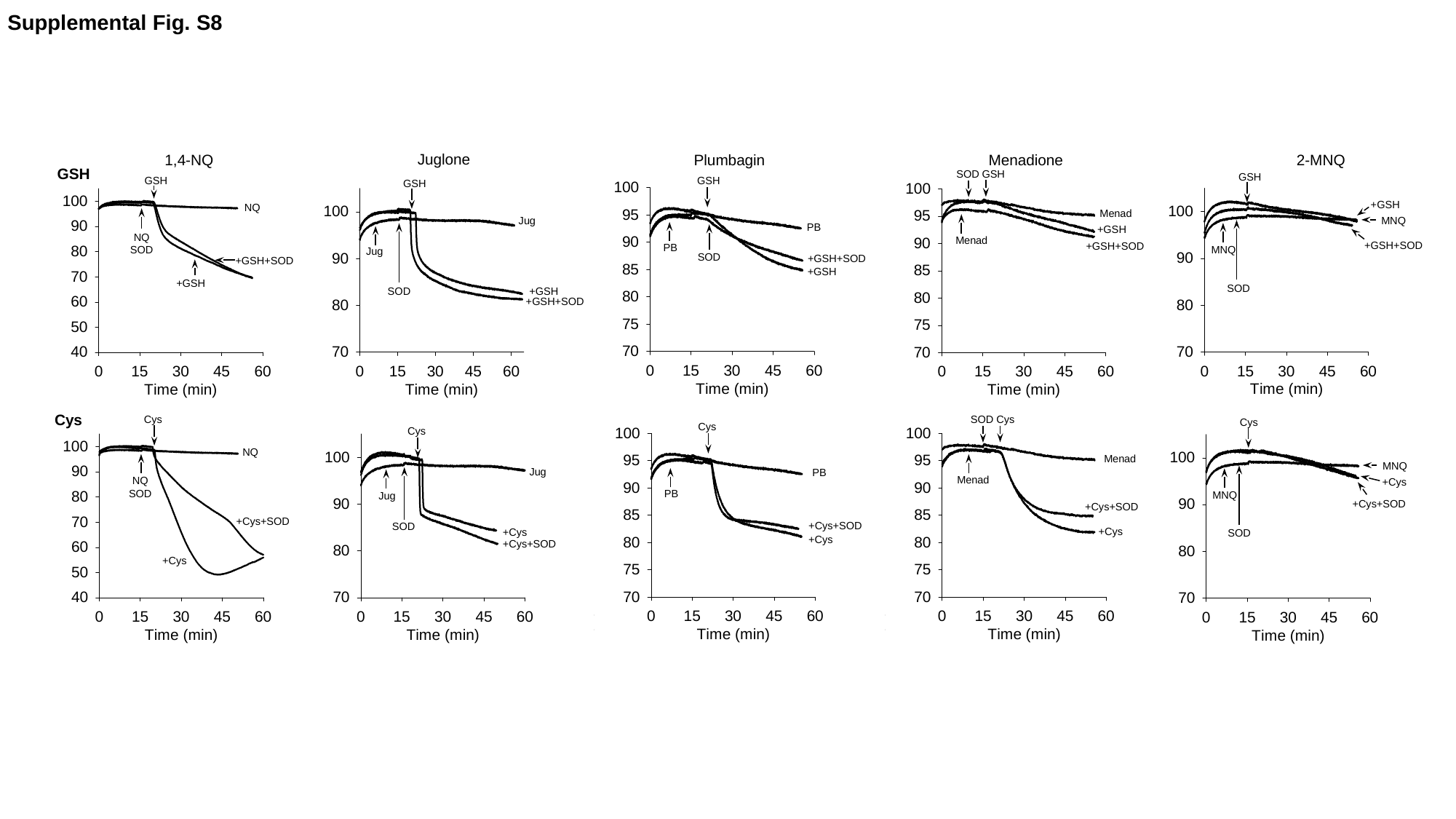

Supplemental Fig. S8
GSH
+GSH
MNQ
+GSH+SOD
MNQ
SOD
GSH
Jug
Jug
+GSH
SOD
+GSH+SOD
Menadione
SOD
GSH
Menad
+GSH
Menad
+GSH+SOD
Juglone
1,4-NQ
GSH
NQ
NQ
SOD
+GSH+SOD
+GSH
Plumbagin
2-MNQ
GSH
GSH
PB
PB
SOD
+GSH+SOD
+GSH
Cys
PB
PB
+Cys+SOD
+Cys
SOD
Cys
Menad
Menad
+Cys+SOD
+Cys
Cys
Jug
Jug
SOD
+Cys
+Cys+SOD
Cys
MNQ
+Cys
MNQ
+Cys+SOD
SOD
Cys
Cys
NQ
NQ
SOD
+Cys+SOD
+Cys

## Slide 9
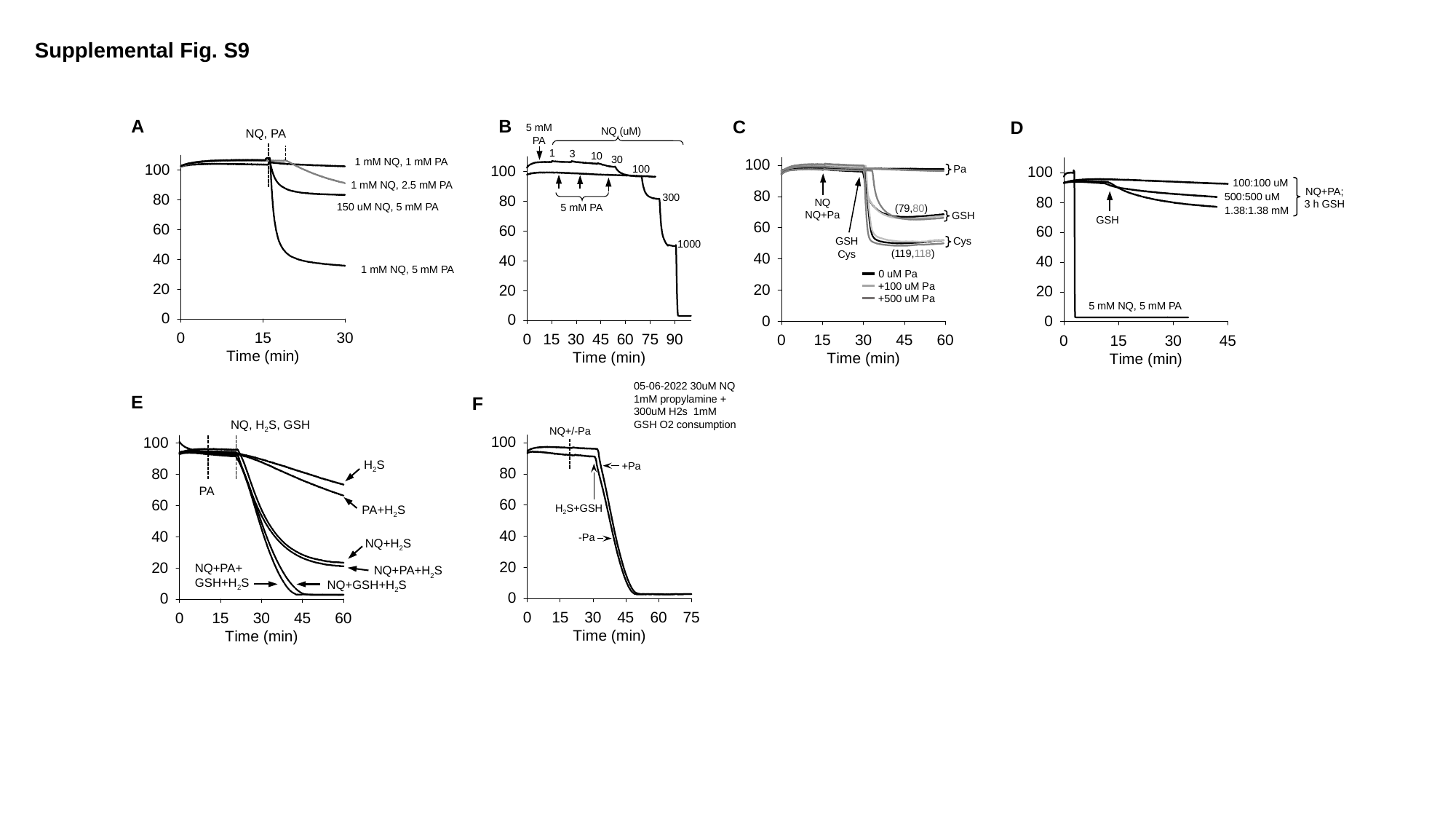

Supplemental Fig. S9
1 mM NQ, 1 mM PA
1 mM NQ, 2.5 mM PA
150 uM NQ, 5 mM PA
1 mM NQ, 5 mM PA
A
5 mM
PA
NQ (uM)
1
3
10
30
100
300
5 mM PA
1000
Pa
NQ
NQ+Pa
GSH
GSH
Cys
Cys
0 uM Pa
+100 uM Pa
+500 uM Pa
(79,80)
(119,118)
100:100 uM
NQ+PA;
3 h GSH
500:500 uM
1.38:1.38 mM
GSH
5 mM NQ, 5 mM PA
B
C
D
NQ, PA
05-06-2022 30uM NQ 1mM propylamine + 300uM H2s 1mM GSH O2 consumption
NQ+/-Pa
+Pa
H2S+GSH
-Pa
H2S
PA+H2S
NQ+H2S
NQ+PA+
GSH+H2S
NQ+PA+H2S
NQ+GSH+H2S
E
F
NQ, H2S, GSH
PA

## Slide 10
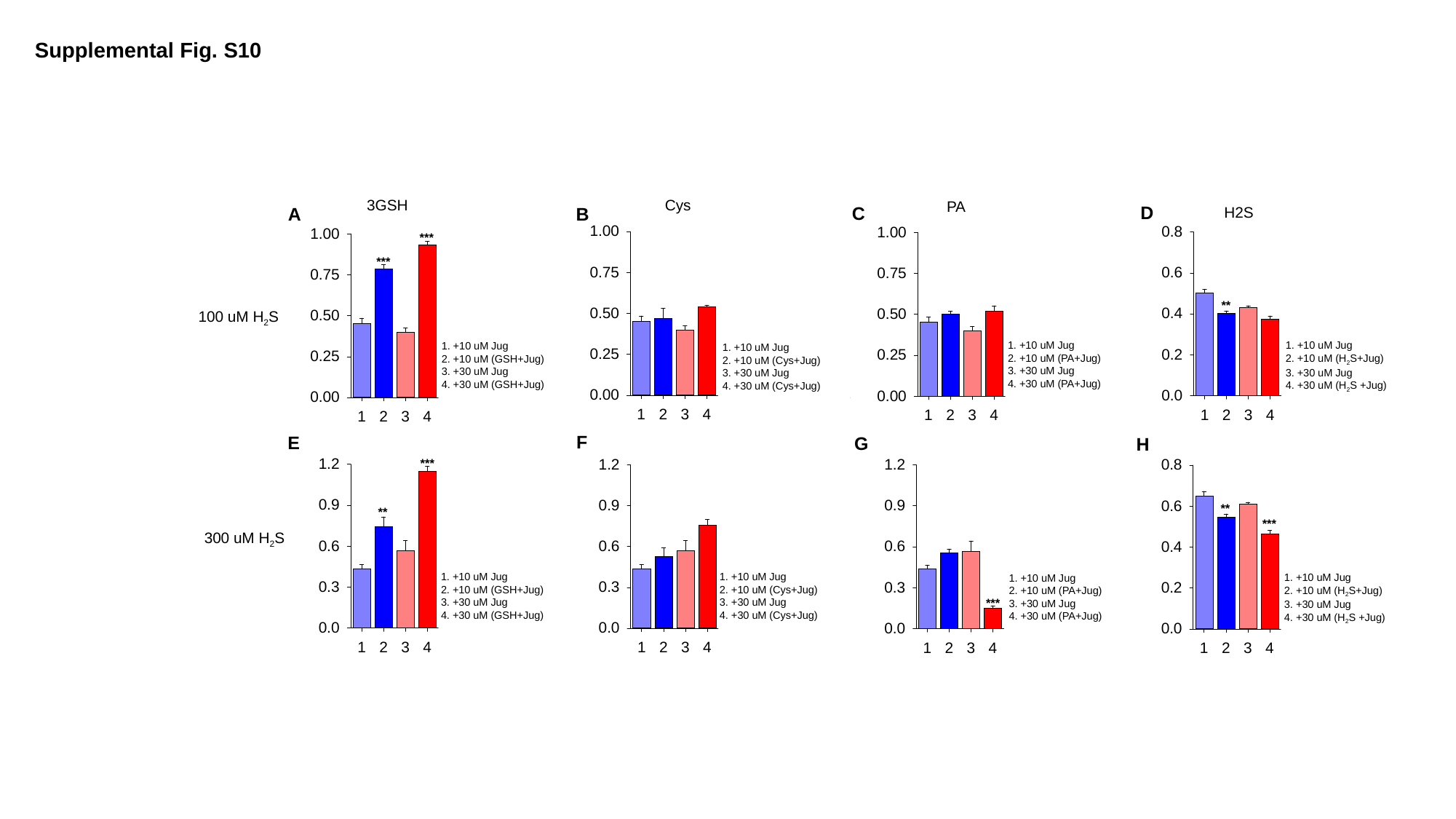

Supplemental Fig. S10
1. +10 uM Jug
2. +10 uM (Cys+Jug)
3. +30 uM Jug
4. +30 uM (Cys+Jug)
1. +10 uM Jug
2. +10 uM (PA+Jug)
3. +30 uM Jug
4. +30 uM (PA+Jug)
***
***
1. +10 uM Jug
2. +10 uM (GSH+Jug)
3. +30 uM Jug
4. +30 uM (GSH+Jug)
Cys
3GSH
PA
D
C
B
H2S
A
**
100 uM H2S
1. +10 uM Jug
2. +10 uM (H2S+Jug)
3. +30 uM Jug
4. +30 uM (H2S +Jug)
***
**
1. +10 uM Jug
2. +10 uM (GSH+Jug)
3. +30 uM Jug
4. +30 uM (GSH+Jug)
1. +10 uM Jug
2. +10 uM (Cys+Jug)
3. +30 uM Jug
4. +30 uM (Cys+Jug)
1. +10 uM Jug
2. +10 uM (PA+Jug)
3. +30 uM Jug
4. +30 uM (PA+Jug)
***
F
E
G
H
**
***
300 uM H2S
1. +10 uM Jug
2. +10 uM (H2S+Jug)
3. +30 uM Jug
4. +30 uM (H2S +Jug)

## Slide 11
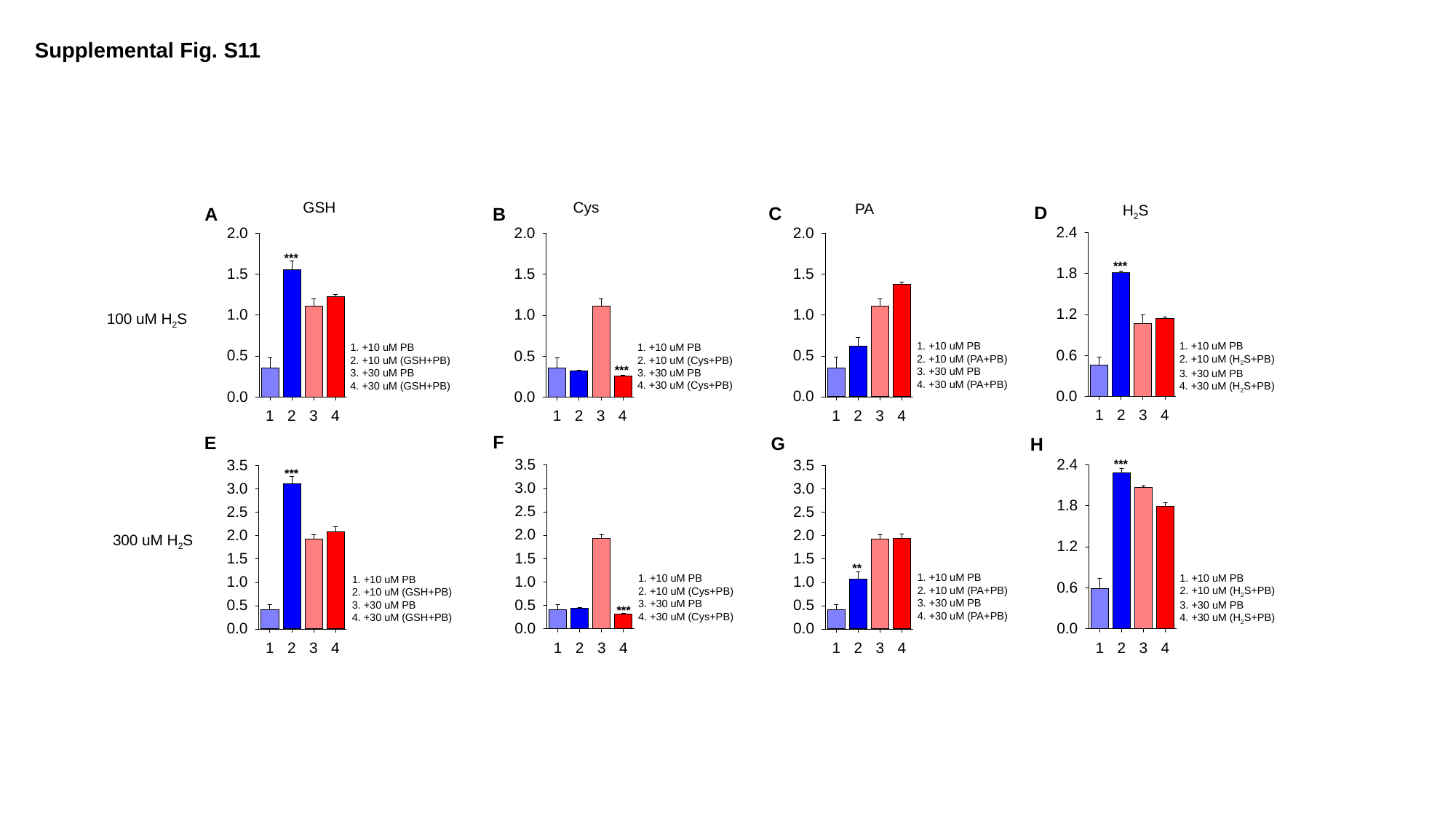

Supplemental Fig. S11
Cys
GSH
PA
H2S
D
C
B
A
***
1. +10 uM PB
2. +10 uM (Cys+PB)
3. +30 uM PB
4. +30 uM (Cys+PB)
***
***
100 uM H2S
1. +10 uM PB
2. +10 uM (H2S+PB)
3. +30 uM PB
4. +30 uM (H2S+PB)
1. +10 uM PB
2. +10 uM (PA+PB)
3. +30 uM PB
4. +30 uM (PA+PB)
1. +10 uM PB
2. +10 uM (GSH+PB)
3. +30 uM PB
4. +30 uM (GSH+PB)
F
E
G
H
***
***
1. +10 uM PB
2. +10 uM (GSH+PB)
3. +30 uM PB
4. +30 uM (GSH+PB)
300 uM H2S
**
1. +10 uM PB
2. +10 uM (Cys+PB)
3. +30 uM PB
4. +30 uM (Cys+PB)
***
1. +10 uM PB
2. +10 uM (PA+PB)
3. +30 uM PB
4. +30 uM (PA+PB)
1. +10 uM PB
2. +10 uM (H2S+PB)
3. +30 uM PB
4. +30 uM (H2S+PB)

## Slide 12
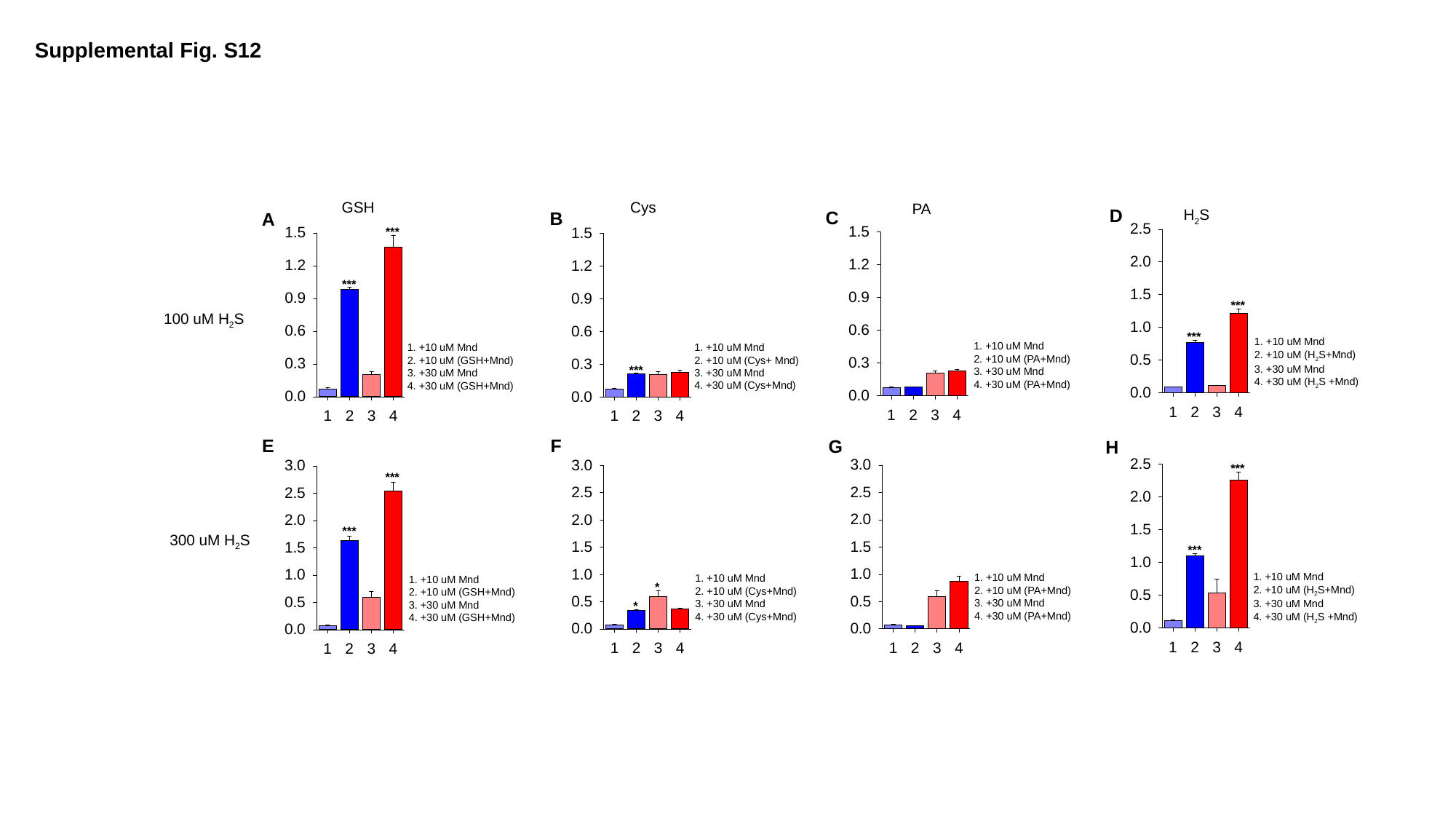

Supplemental Fig. S12
Cys
GSH
PA
D
H2S
C
B
A
***
***
***
100 uM H2S
***
1. +10 uM Mnd
2. +10 uM (H2S+Mnd)
3. +30 uM Mnd
4. +30 uM (H2S +Mnd)
1. +10 uM Mnd
2. +10 uM (PA+Mnd)
3. +30 uM Mnd
4. +30 uM (PA+Mnd)
1. +10 uM Mnd
2. +10 uM (Cys+ Mnd)
3. +30 uM Mnd
4. +30 uM (Cys+Mnd)
***
1. +10 uM Mnd
2. +10 uM (GSH+Mnd)
3. +30 uM Mnd
4. +30 uM (GSH+Mnd)
F
E
G
H
***
***
***
1. +10 uM Mnd
2. +10 uM (Cys+Mnd)
3. +30 uM Mnd
4. +30 uM (Cys+Mnd)
*
300 uM H2S
***
1. +10 uM Mnd
2. +10 uM (H2S+Mnd)
3. +30 uM Mnd
4. +30 uM (H2S +Mnd)
1. +10 uM Mnd
2. +10 uM (PA+Mnd)
3. +30 uM Mnd
4. +30 uM (PA+Mnd)
*
1. +10 uM Mnd
2. +10 uM (GSH+Mnd)
3. +30 uM Mnd
4. +30 uM (GSH+Mnd)
*

## Slide 13
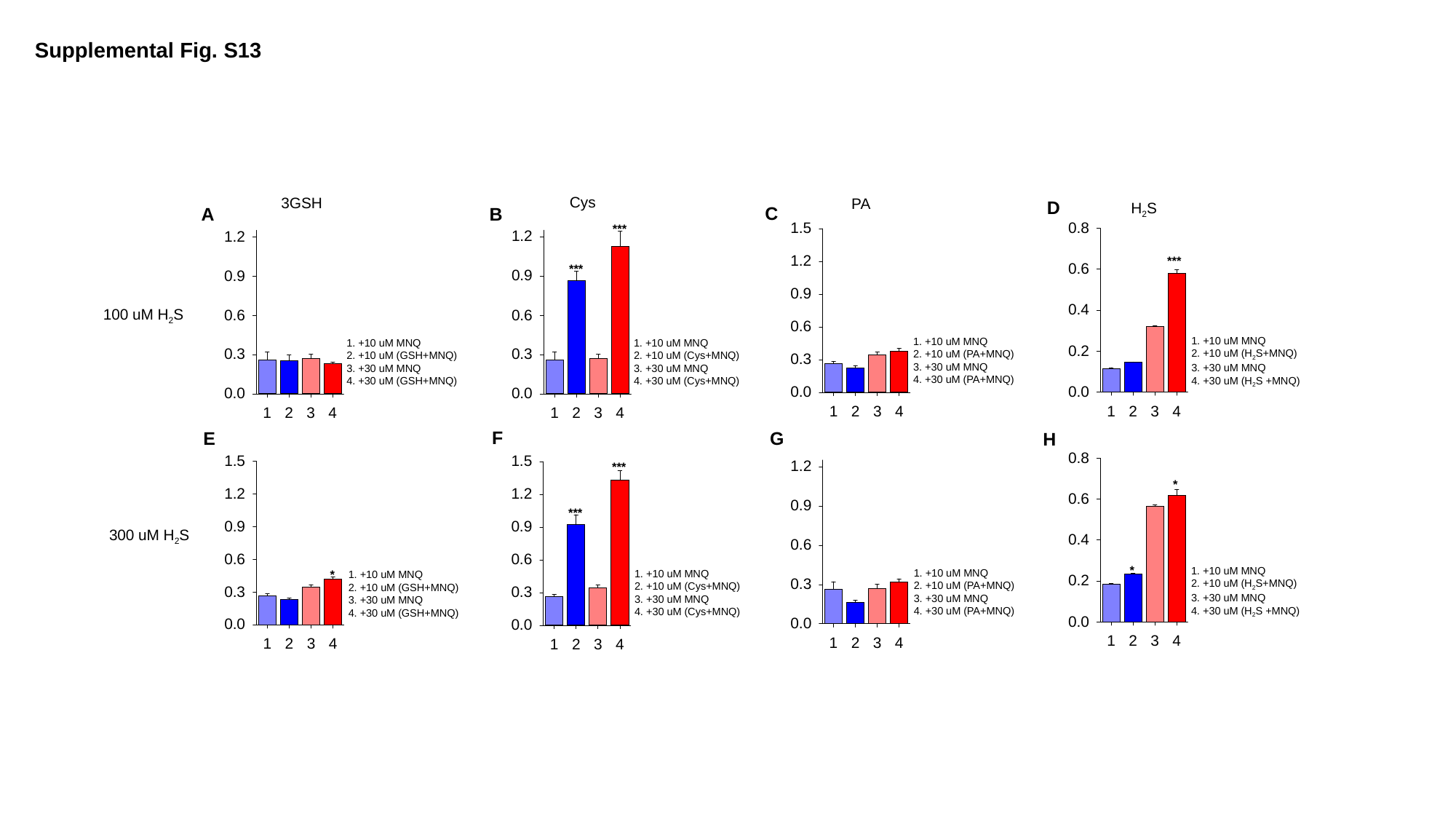

Supplemental Fig. S13
1. +10 uM MNQ
2. +10 uM (PA+MNQ)
3. +30 uM MNQ
4. +30 uM (PA+MNQ)
***
***
1. +10 uM MNQ
2. +10 uM (Cys+MNQ)
3. +30 uM MNQ
4. +30 uM (Cys+MNQ)
1. +10 uM MNQ
2. +10 uM (GSH+MNQ)
3. +30 uM MNQ
4. +30 uM (GSH+MNQ)
Cys
3GSH
PA
D
H2S
C
B
A
***
100 uM H2S
1. +10 uM MNQ
2. +10 uM (PA+MNQ)
3. +30 uM MNQ
4. +30 uM (PA+MNQ)
*
1. +10 uM MNQ
2. +10 uM (GSH+MNQ)
3. +30 uM MNQ
4. +30 uM (GSH+MNQ)
***
***
1. +10 uM MNQ
2. +10 uM (Cys+MNQ)
3. +30 uM MNQ
4. +30 uM (Cys+MNQ)
F
E
G
H
*
300 uM H2S
*
1. +10 uM MNQ
2. +10 uM (H2S+MNQ)
3. +30 uM MNQ
4. +30 uM (H2S +MNQ)
1. +10 uM MNQ
2. +10 uM (H2S+MNQ)
3. +30 uM MNQ
4. +30 uM (H2S +MNQ)

## Slide 14
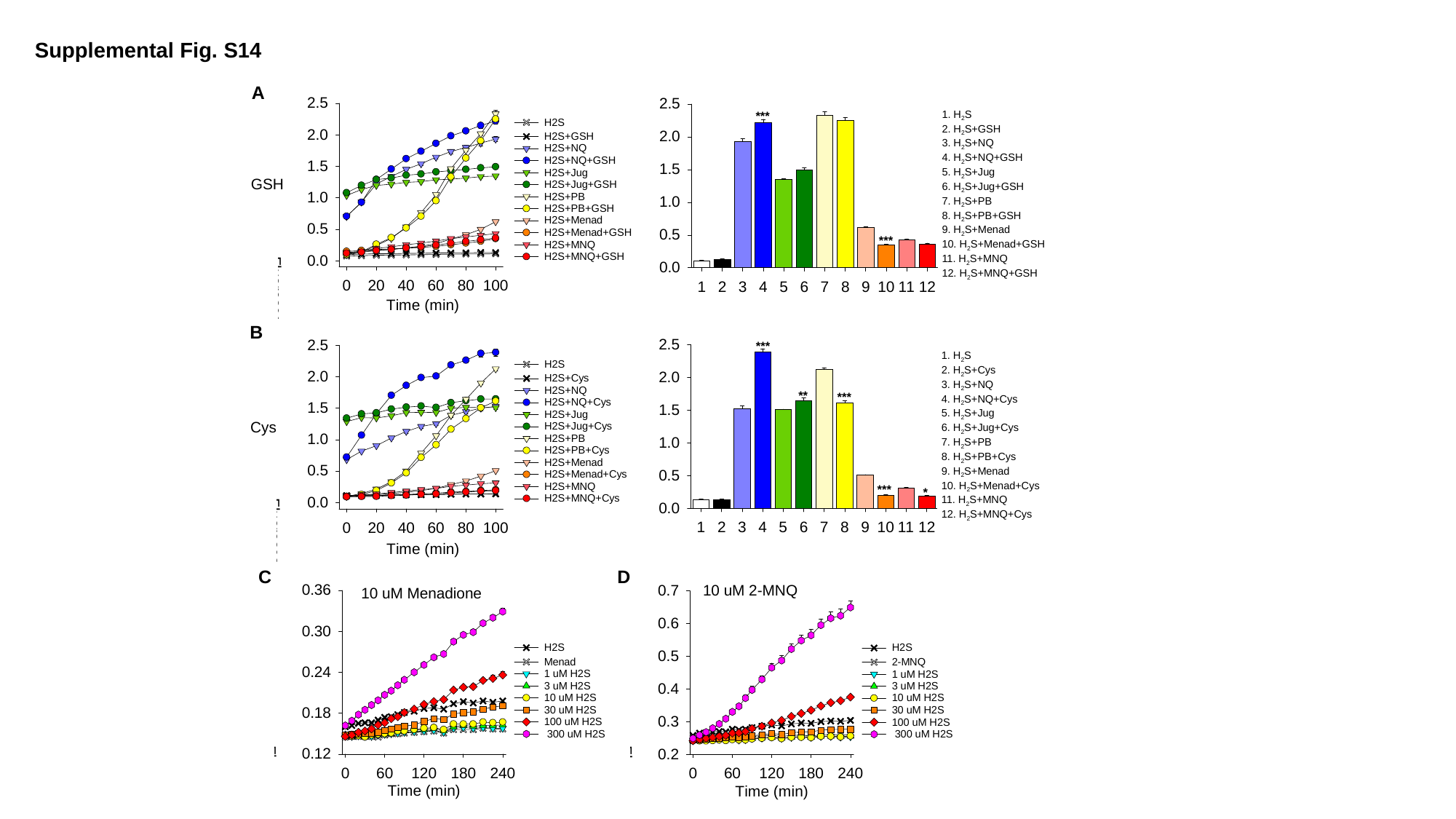

Supplemental Fig. S14
1. H2S
2. H2S+GSH
3. H2S+NQ
4. H2S+NQ+GSH
5. H2S+Jug
6. H2S+Jug+GSH
7. H2S+PB
8. H2S+PB+GSH
9. H2S+Menad
10. H2S+Menad+GSH
11. H2S+MNQ
12. H2S+MNQ+GSH
***
GSH
***
***
**
***
***
*
1. H2S
2. H2S+Cys
3. H2S+NQ
4. H2S+NQ+Cys
5. H2S+Jug
6. H2S+Jug+Cys
7. H2S+PB
8. H2S+PB+Cys
9. H2S+Menad
10. H2S+Menad+Cys
11. H2S+MNQ
12. H2S+MNQ+Cys
Cys
A
B
C
D
10 uM 2-MNQ
10 uM Menadione

## Slide 15
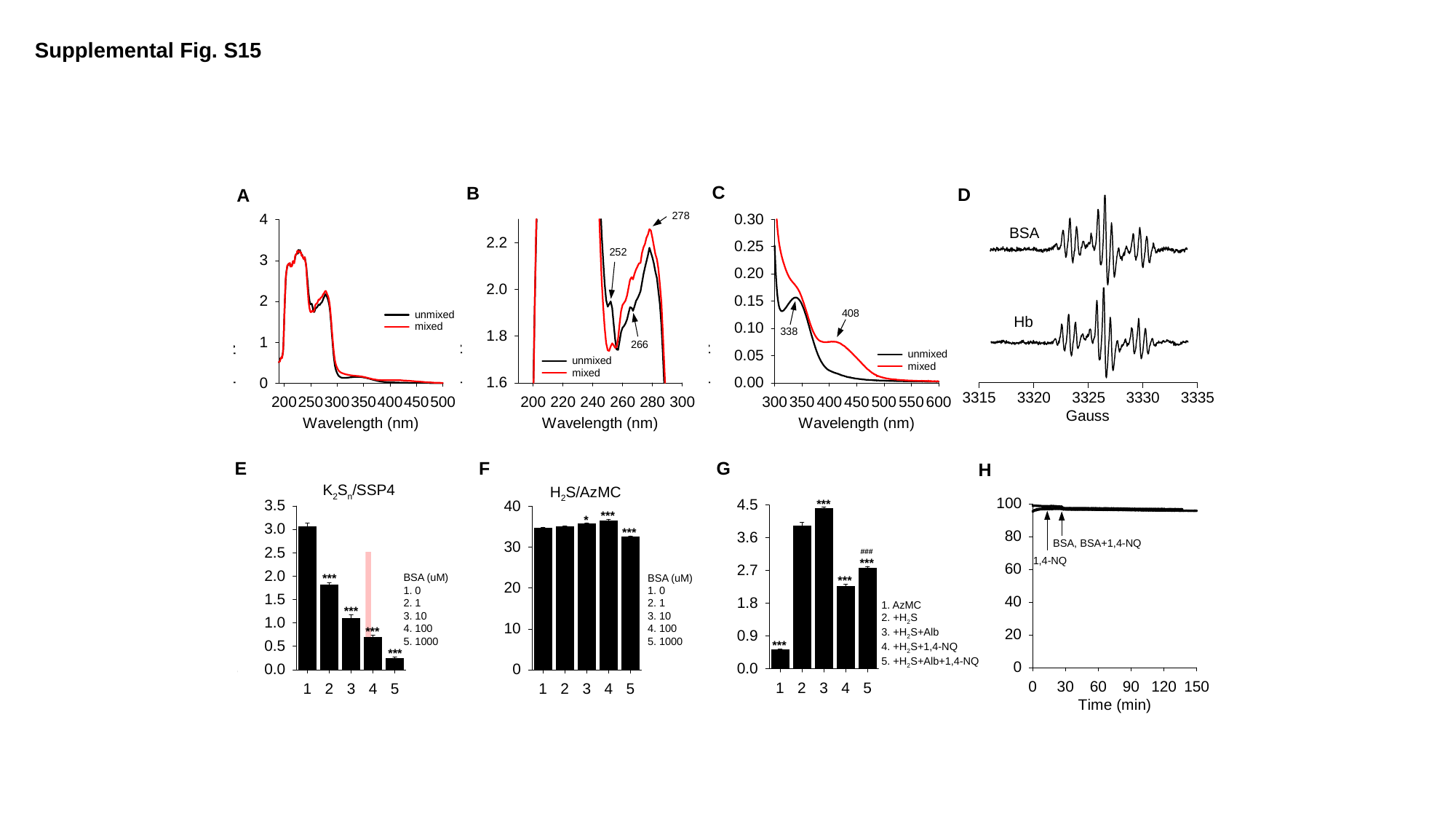

Supplemental Fig. S15
D
BSA
Hb
3315
3320
3325
3330
3335
Gauss
278
252
266
408
338
C
B
A
G
***
***
***
1. AzMC
2. +H2S
3. +H2S+Alb
4. +H2S+1,4-NQ
5. +H2S+Alb+1,4-NQ
K2Sn/SSP4
BSA (uM)
1. 0
2. 1
3. 10
4. 100
5. 1000
H2S/AzMC
BSA (uM)
1. 0
2. 1
3. 10
4. 100
5. 1000
***
*
***
E
F
***
***
***
***
***
H
BSA, BSA+1,4-NQ
1,4-NQ
###
